# Short-term health-related quality of life and pain after total-port robotic versus uniportal video-assisted thoracoscopic anatomical lung resection: a matched prospective cohort study

**DOI:** 10.3389/fsurg.2026.1919293

**Published:** 2026-10-28

**Authors:** Xinghaobo Shi, Miao He, Ping Zhang, Hui Tao, Fengchen Du, Yang Yang, Shenyan Zheng, Hui Chen

**Affiliations:** Department of Thoracic Surgery, Shanghai Chest Hospital, Shanghai Jiao Tong University School of Medicine, Shanghai, China

**Keywords:** health-related quality of life, lobectomy, pain, patient-reported outcomes, robotic-assisted thoracoscopic surgery, segmentectomy, uniportal VATS

## Abstract

**Background:**

Direct comparisons of patient-reported recovery after total-port robotic-assisted thoracoscopic surgery (RATS) and uniportal video-assisted thoracoscopic surgery (VATS) are limited. We compared short-term health-related quality of life (HRQoL) and pain after anatomical lung resection performed with the two approaches.

**Methods:**

This single-centre prospective observational cohort included consecutive patients undergoing lung surgery from January 2025 to June 2026. The revision analysis was restricted to single lobectomy or segmentectomy. VATS controls were limited to treating teams that used both approaches. RATS and VATS cases were matched 1:1 within resection type and lymph-node management using a propensity model that included treating team, demographic, pulmonary-function and CT tumour variables. HRQoL and pain were assessed preoperatively, on postoperative day (POD) 1, and at 1 and 3 months.

**Results:**

Thirty-five RATS patients were matched to 35 VATS patients, including 14 lobectomy pairs and 21 segmentectomy pairs. Global HRQoL was not significantly different between groups at baseline (77.4 vs. 81.0), POD 1 (69.3 vs. 71.0), 1 month (86.6 vs. 85.1), or 3 months (84.4 vs. 88.5; all *P* > 0.05). NRS pain was similar on POD 1 (2.2 vs. 2.2) and at 3 months (0.3 vs. 0.0); at 1 month, RATS had a small nominally higher score (0.9 vs. 0.4, *P* = 0.018). RATS had longer operative time and greater total drainage, whereas chest-tube duration, complication rates and systematic lymph-node dissection were not significantly different.

**Conclusions:**

In this small procedure- and nodal-management-matched cohort, the observed short-term HRQoL and pain trajectories were broadly similar. The estimates are imprecise and do not establish equivalence between the surgical approaches. Larger studies comparing the same operations with surgeon-level adjustment are required.

## 1 Introduction

Lung cancer remains the most frequently diagnosed malignancy and the leading cause of cancer-related death worldwide, accounting for almost 2.5 million new cases in 2022 (1). For early-stage non-small-cell lung cancer, anatomical pulmonary resection with systematic lymph-node evaluation remains a cornerstone of curative treatment (2). For small peripheral tumours, segmentectomy has been established as a valid parenchyma-sparing alternative to lobectomy in randomized trials (28). Recent randomized evidence further supports sublobar resection for selected peripheral stage IA tumours (29). Minimally invasive techniques have progressively displaced thoracotomy because they reduce surgical trauma and accelerate recovery while preserving oncological efficacy (3). Uniportal VATS represents the most condensed thoracoscopic configuration and has shown favourable perioperative outcomes without compromising long-term survival (4). The uniportal VATS technique was pioneered and refined over the past decade (22).

Uniportal VATS nevertheless involves instrument crowding through one incision, rigid non-articulated instruments and a two-dimensional view. The da Vinci robotic platform provides tremor-filtered wristed instrumentation, magnified three-dimensional vision and improved ergonomics (5). Large database analyses suggest comparable perioperative outcomes between robotic and thoracoscopic approaches (23). Long-term survival after robotic lobectomy appears similar to that after VATS and thoracotomy (24). A meta-analysis of early robotic lobectomy series reported acceptable feasibility and safety (25). Robotic-assisted surgery may offer particular benefit in older patients with resectable lung cancer (26). Nationwide data confirm increasing adoption of robotic lobectomy with perioperative outcomes comparable to VATS (27). Robotic lung surgery may use a utility incision or a completely portal configuration (6). The latter uses trocar incisions without a utility thoracotomy and has matured since its introduction around 2010 (7–11).

HRQoL is a central patient-reported outcome in thoracic oncology (12). The EORTC QLQ-C30 and QLQ-LC13 are validated instruments for patients with lung cancer (13, 14). The RVlob randomized trial found no material difference in HRQoL or pain between robotic-assisted and video-assisted lobectomy (15), whereas early RAVAL results suggested comparable health utility and acceptable cost-effectiveness (16, 17). Most evidence, however, compares robotic surgery with multiportal VATS rather than contemporary uniportal VATS.

We therefore conducted a prospective observational cohort study to describe short-term patient-reported HRQoL and pain after total-port RATS vs. uniportal VATS. In response to peer review, the comparative analysis was restricted to matched lobectomy and segmentectomy cases with the same lymph-node-management category, so that differences in operative extent did not define the comparison.

## 2 Materials and methods

### 2.1 Study design and participants

This single-centre, prospective, non-interventional observational cohort was conducted in the Department of Thoracic Surgery, Shanghai Chest Hospital, between January 2025 and June 2026. Consecutive adults undergoing total-port RATS or uniportal VATS were screened. Eligibility required the ability to complete the questionnaires and written informed consent. Open thoracotomy, conversion before completion of the target procedure, uncoded or non-target approaches, and incomplete baseline assessment were excluded. The protocol was approved by the Ethics Committee of Shanghai Chest Hospital (approval No. IS25060; 25 March 2025), and all participants provided written informed consent.

### 2.2 Surgical techniques and treating teams

Uniportal VATS was performed through one 3–4 cm incision without rib spreading. Total-port RATS used four robotic arms without a utility incision. The extent of resection and lymph-node management were determined from imaging, frozen-section findings and oncological principles. The prospective database recorded the treating surgical team, not the individual operating surgeon. Three treating teams performed anatomical resections with RATS during the study period and also had VATS cases available for common-support matching. Individual surgeon identity and surgeon-specific experience were not captured.

### 2.3 Patient-reported outcome instruments

The EORTC QLQ-C30 (version 3) measures five functional scales, global health status/QoL, three symptom scales and six single symptoms, including insomnia (18). The validated QLQ-LC13 module includes cough, haemoptysis, dyspnoea, treatment-related symptoms (including alopecia) and site-specific pain (19). Alopecia was retained because the complete validated module was administered; it was not selected as a surgery-specific endpoint. Scores were transformed to 0–100 according to the EORTC manual (30). Pain was also rated on a 0–10 numerical rating scale (NRS), and general health on the EQ-5D-5L visual analogue scale (EQ-VAS) (20, 21). Assessments occurred preoperatively, on POD 1, and at approximately 1 and 3 months.

### 2.4 Outcomes

The patient-reported domains of principal interest were global HRQoL and NRS pain. Secondary outcomes comprised QLQ-C30 and QLQ-LC13 domains, EQ-VAS and perioperative outcomes. The study was exploratory and was not designed as an equivalence or non-inferiority trial.

### 2.5 Postoperative complication definitions

Postoperative events were recorded prospectively in structured and free-text fields. During the revision audit, free-text thoracic events were recoded as recorded air leak, subcutaneous emphysema, atelectasis, chylothorax or other thoracic events. Severity was classified using the Clavien-Dindo system (31). Chest-tube duration longer than 5 days was reported separately as prolonged drainage; it was not assumed to represent prolonged air leak unless air leak was explicitly recorded.

### 2.6 Matching and statistical analysis

To make the compared operations clinically commensurate, the revision analysis excluded wedge, sleeve, combined and non-parenchymal procedures and retained only single lobectomy or segmentectomy. VATS candidates were restricted to treating teams with RATS cases. A logistic propensity model included age, sex, body-mass index, smoking, hypertension, diabetes, chronic obstructive pulmonary disease, ASA class, predicted FEV1, DLCO, CT lesion size, CT solid-component size, pure ground-glass opacity and treating team. Optimal 1:1 matching without replacement was performed within exact strata of resection type and lymph-node-management category using a caliper of 0.25 SD of the logit propensity score. Clinical stage was not separately recorded in the available prospective database; pathological stage was not included because it was determined after resection. Residual confounding was therefore expected and assessed with standardized mean differences.

Continuous perioperative variables are presented as median (interquartile range) and compared with the Mann–Whitney *U*-test. Patient-reported scores are presented as mean ± SD to retain the conventional EORTC scale presentation and compared with the Mann–Whitney *U*-test. Categorical variables are reported as n (%) and compared with Fisher's exact or chi-square tests. All tests were two-sided. No multiplicity correction was applied; secondary findings are hypothesis-generating. Analyses used Python 3.12 (pandas, SciPy and scikit-learn).

## 3 Results

### 3.1 Cohort and matching

Among 1,051 screened records, 999 involved total-port RATS or uniportal VATS. Restriction to single anatomical pulmonary resection yielded 53 RATS and 495 VATS cases. Restriction to the three treating teams with common support left 53 RATS and 145 VATS candidates. Procedure- and nodal-management-exact propensity matching produced 35 pairs: 14 lobectomy pairs and 21 segmentectomy pairs (Figure 1). The matched team distribution was Team A, 6 vs. 4; Team B, 0 vs. 1; and Team C, 29 vs. 30 for RATS and VATS, respectively. Most measured baseline variables were similar, although residual imbalance remained (maximum absolute standardized mean difference 0.295; Table 1).

**Figure 1:**
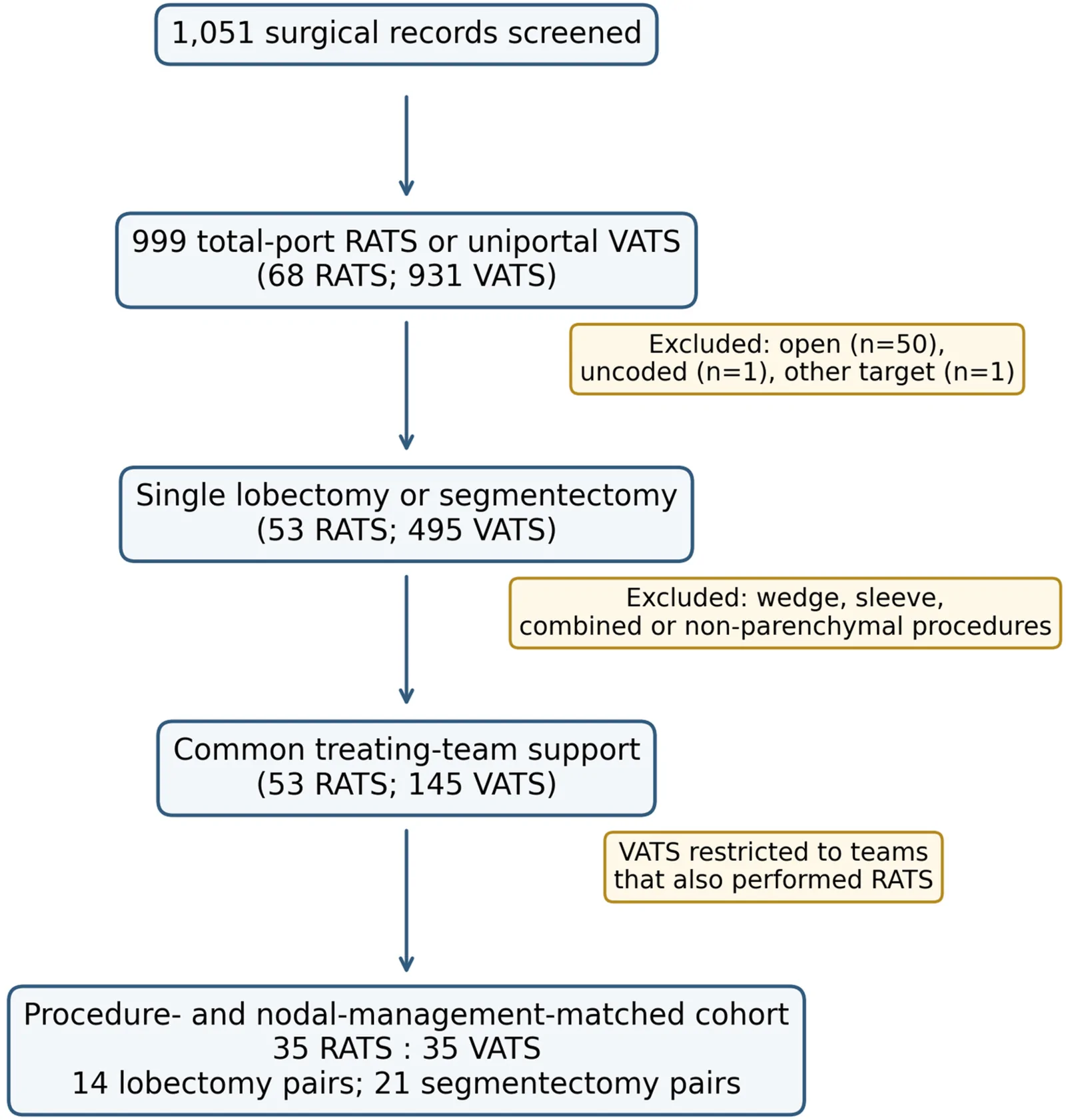
Participant flow and revision matching strategy. RATS, total-port robotic-assisted thoracoscopic surgery; VATS, uniportal video-assisted thoracoscopic surgery.

**Table 1:** Baseline characteristics of the matched cohort.

Variable	RATS (*n* = 35)	VATS (*n* = 35)	**P**	**SMD**
Age, years	57.0 ± 10.1	56.7 ± 11.0	0.948	0.024
Female sex	19 (54.3%)	22 (62.9%)	0.467	0.175
BMI, kg/m²	23.8 ± 2.3	24.2 ± 3.1	0.660	0.163
Smoking history	11 (31.4%)	8 (22.9%)	0.420	0.194
Hypertension	9 (25.7%)	11 (31.4%)	0.597	0.127
Diabetes mellitus	3 (8.6%)	2 (5.7%)	>0.99	0.111
COPD	0 (0.0%)	0 (0.0%)	>0.99	0.0
FEV1, % predicted	97.1 ± 15.7	98.0 ± 14.3	0.897	0.061
DLCO, % predicted	103.3 ± 17.2	108.7 ± 19.2	0.414	0.295
CT lesion size, mm	8.4 ± 9.1	8.5 ± 8.7	0.894	0.005
CT solid-component size, mm	6.9 ± 10.4	6.8 ± 9.2	0.941	0.017
Pure GGO	2 (5.7%)	1 (2.9%)	>0.99	0.141
ASA class 1	1 (2.9%)	0 (0.0%)	>0.99	0.243
ASA class 2	26 (74.3%)	27 (77.1%)	0.780	0.067
ASA class 3	8 (22.9%)	8 (22.9%)	>0.99	0.0

Data are mean ± SD or *n* (%). SMD values are absolute. Clinical stage was not available as a separate preoperative variable. BMI, body-mass index; COPD, chronic obstructive pulmonary disease; FEV1, forced expiratory volume in 1 s; DLCO, diffusing capacity for carbon monoxide; GGO, ground-glass opacity; ASA, American Society of Anesthesiologists.

### 3.2 Perioperative outcomes and complications

Because resection type and lymph-node-management category were matched exactly, both groups contained 14 lobectomies, 21 segmentectomies and 34 systematic lymph-node dissections. RATS had longer operative time (median 168 vs. 110 min, *P* < 0.001) and greater total drainage (510 vs. 370 mL, *P* = 0.042). Chest-tube duration (4 vs. 3 days, *P* = 0.154) and postoperative hospital stay (3 vs. 3 days, *P* = 0.068) were not significantly different. In the resection-specific panels, the drainage difference was concentrated in segmentectomy (Table 2).

**Table 2:** Perioperative outcomes overall and by matched resection type.

**Panel**	**Outcome**	**RATS**	**VATS**	**P**
Overall procedure-matched cohort (*n* = 35 per group)	Operative time, min	168.0 (135.0–212.5)	110.0 (89.0–132.5)	<0.001
Overall procedure-matched cohort (*n* = 35 per group)	Estimated blood loss, mL	50.0 (50.0–50.0)	50.0 (50.0–50.0)	0.424
Overall procedure-matched cohort (*n* = 35 per group)	Chest-tube duration, d	4.0 (3.0–4.8)	3.0 (2.2–4.0)	0.154
Overall procedure-matched cohort (*n* = 35 per group)	Total drainage, mL	510.0 (330.0–632.5)	370.0 (115.0–540.0)	0.042
Overall procedure-matched cohort (*n* = 35 per group)	Postoperative hospital stay, d	3.0 (3.0–4.0)	3.0 (2.0–3.0)	0.068
Overall procedure-matched cohort (*n* = 35 per group)	ICU stay, d	0.0 (0.0–0.0)	0.0 (0.0–0.0)	0.339
Overall procedure-matched cohort (*n* = 35 per group)	Systematic lymph-node dissection	34 (97.1%)	34 (97.1%)	>0.99
Overall procedure-matched cohort (*n* = 35 per group)	Any postoperative complication	3 (8.6%)	2 (5.9%)	>0.99
Overall procedure-matched cohort (*n* = 35 per group)	Any thoracic complication	3 (8.6%)	2 (5.7%)	>0.99
Overall procedure-matched cohort (*n* = 35 per group)	Recorded air leak	2 (5.7%)	1 (2.9%)	>0.99
Overall procedure-matched cohort (*n* = 35 per group)	Prolonged drainage (>5 d)	5 (14.3%)	3 (8.6%)	0.710
Overall procedure-matched cohort (*n* = 35 per group)	Clavien-Dindo grade I	3 (8.6%)	2 (5.7%)	>0.99
Overall procedure-matched cohort (*n* = 35 per group)	Clavien-Dindo grade II	0 (0.0%)	0 (0.0%)	>0.99
Overall procedure-matched cohort (*n* = 35 per group)	Clavien-Dindo grade IIIa	0 (0.0%)	0 (0.0%)	>0.99
Overall procedure-matched cohort (*n* = 35 per group)	Conversion to thoracotomy	0 (0.0%)	0 (0.0%)	>0.99
Overall procedure-matched cohort (*n* = 35 per group)	Reoperation	0 (0.0%)	0 (0.0%)	>0.99
Lobectomy stratum (*n* = 14 per group)	Operative time, min	157.5 (134.8–213.8)	122.5 (108.5–142.5)	0.063
Lobectomy stratum (*n* = 14 per group)	Estimated blood loss, mL	50.0 (50.0–50.0)	50.0 (50.0–50.0)	0.306
Lobectomy stratum (*n* = 14 per group)	Chest-tube duration, d	4.0 (3.0–5.0)	3.0 (3.0–5.0)	0.854
Lobectomy stratum (*n* = 14 per group)	Total drainage, mL	585.0 (405.0–815.0)	540.0 (420.0–880.0)	0.865
Lobectomy stratum (*n* = 14 per group)	Postoperative hospital stay, d	4.0 (3.0–5.8)	3.0 (3.0–4.0)	0.486
Lobectomy stratum (*n* = 14 per group)	ICU stay, d	0.0 (0.0–0.0)	0.0 (0.0–0.0)	0.374
Lobectomy stratum (*n* = 14 per group)	Systematic lymph-node dissection	14 (100.0%)	14 (100.0%)	>0.99
Lobectomy stratum (*n* = 14 per group)	Any postoperative complication	2 (14.3%)	2 (14.3%)	>0.99
Lobectomy stratum (*n* = 14 per group)	Any thoracic complication	2 (14.3%)	2 (14.3%)	>0.99
Lobectomy stratum (*n* = 14 per group)	Recorded air leak	1 (7.1%)	1 (7.1%)	>0.99
Lobectomy stratum (*n* = 14 per group)	Prolonged drainage (>5 d)	3 (21.4%)	3 (21.4%)	>0.99
Lobectomy stratum (*n* = 14 per group)	Clavien-Dindo grade I	2 (14.3%)	2 (14.3%)	>0.99
Lobectomy stratum (*n* = 14 per group)	Clavien-Dindo grade II	0 (0.0%)	0 (0.0%)	>0.99
Lobectomy stratum (*n* = 14 per group)	Clavien-Dindo grade IIIa	0 (0.0%)	0 (0.0%)	>0.99
Lobectomy stratum (*n* = 14 per group)	Conversion to thoracotomy	0 (0.0%)	0 (0.0%)	>0.99
Lobectomy stratum (*n* = 14 per group)	Reoperation	0 (0.0%)	0 (0.0%)	>0.99
Segmentectomy stratum (*n* = 21 per group)	Operative time, min	180.0 (137.0–212.0)	110.0 (81.0–125.0)	<0.001
Segmentectomy stratum (*n* = 21 per group)	Estimated blood loss, mL	50.0 (50.0–50.0)	50.0 (50.0–50.0)	>0.99
Segmentectomy stratum (*n* = 21 per group)	Chest-tube duration, d	3.0 (3.0–4.0)	3.0 (2.0–4.0)	0.094
Segmentectomy stratum (*n* = 21 per group)	Total drainage, mL	420.0 (300.0–610.0)	250.0 (100.0–410.0)	0.003
Segmentectomy stratum (*n* = 21 per group)	Postoperative hospital stay, d	3.0 (3.0–4.0)	3.0 (2.0–3.0)	0.060
Segmentectomy stratum (*n* = 21 per group)	ICU stay, d	0.0 (0.0–0.0)	0.0 (0.0–0.0)	>0.99
Segmentectomy stratum (*n* = 21 per group)	Systematic lymph-node dissection	20 (95.2%)	20 (95.2%)	>0.99
Segmentectomy stratum (*n* = 21 per group)	Any postoperative complication	1 (4.8%)	0 (0.0%)	>0.99
Segmentectomy stratum (*n* = 21 per group)	Any thoracic complication	1 (4.8%)	0 (0.0%)	>0.99
Segmentectomy stratum (*n* = 21 per group)	Recorded air leak	1 (4.8%)	0 (0.0%)	>0.99
Segmentectomy stratum (*n* = 21 per group)	Prolonged drainage (>5 d)	2 (9.5%)	0 (0.0%)	0.488
Segmentectomy stratum (*n* = 21 per group)	Clavien-Dindo grade I	1 (4.8%)	0 (0.0%)	>0.99
Segmentectomy stratum (*n* = 21 per group)	Clavien-Dindo grade II	0 (0.0%)	0 (0.0%)	>0.99
Segmentectomy stratum (*n* = 21 per group)	Clavien-Dindo grade IIIa	0 (0.0%)	0 (0.0%)	>0.99
Segmentectomy stratum (*n* = 21 per group)	Conversion to thoracotomy	0 (0.0%)	0 (0.0%)	>0.99
Segmentectomy stratum (*n* = 21 per group)	Reoperation	0 (0.0%)	0 (0.0%)	>0.99

*C*ontinuous data are median (interquartile range); categorical data are *n* (%). Denominators vary where source fields were missing. Thoracic events may overlap. Prolonged drainage (>5 d) was not equated with air leak unless air leak was explicitly recorded.

Any postoperative complication occurred in 3 RATS and 2 VATS patients. Thoracic events occurred in 3 (8.6%) and 2 (5.7%), including recorded air leak in 2 and 1, subcutaneous emphysema in 1 and 1, and chylothorax in 1 and 0, respectively. Some patients had more than one recorded event. All events were Clavien-Dindo grade I; no grade II or higher complication, conversion or reoperation occurred in the matched cohort. Prolonged drainage occurred in 5 (14.3%) and 3 (8.6%) patients (*P* = 0.710).

### 3.3 HRQoL and functional recovery

Global HRQoL did not differ significantly at baseline (77.4 vs. 81.0, *P* = 0.923), POD 1 (69.3 vs. 71.0, *P* = 0.952), 1 month (86.6 vs. 85.1, *P* = 0.699) or 3 months (84.4 vs. 88.5, *P* = 0.227). Both groups showed transient deterioration on POD 1 and recovery by 1 month (Figure 2). Physical, role, emotional and social functioning followed similar descriptive trajectories (Table 3).

**Figure 2:**
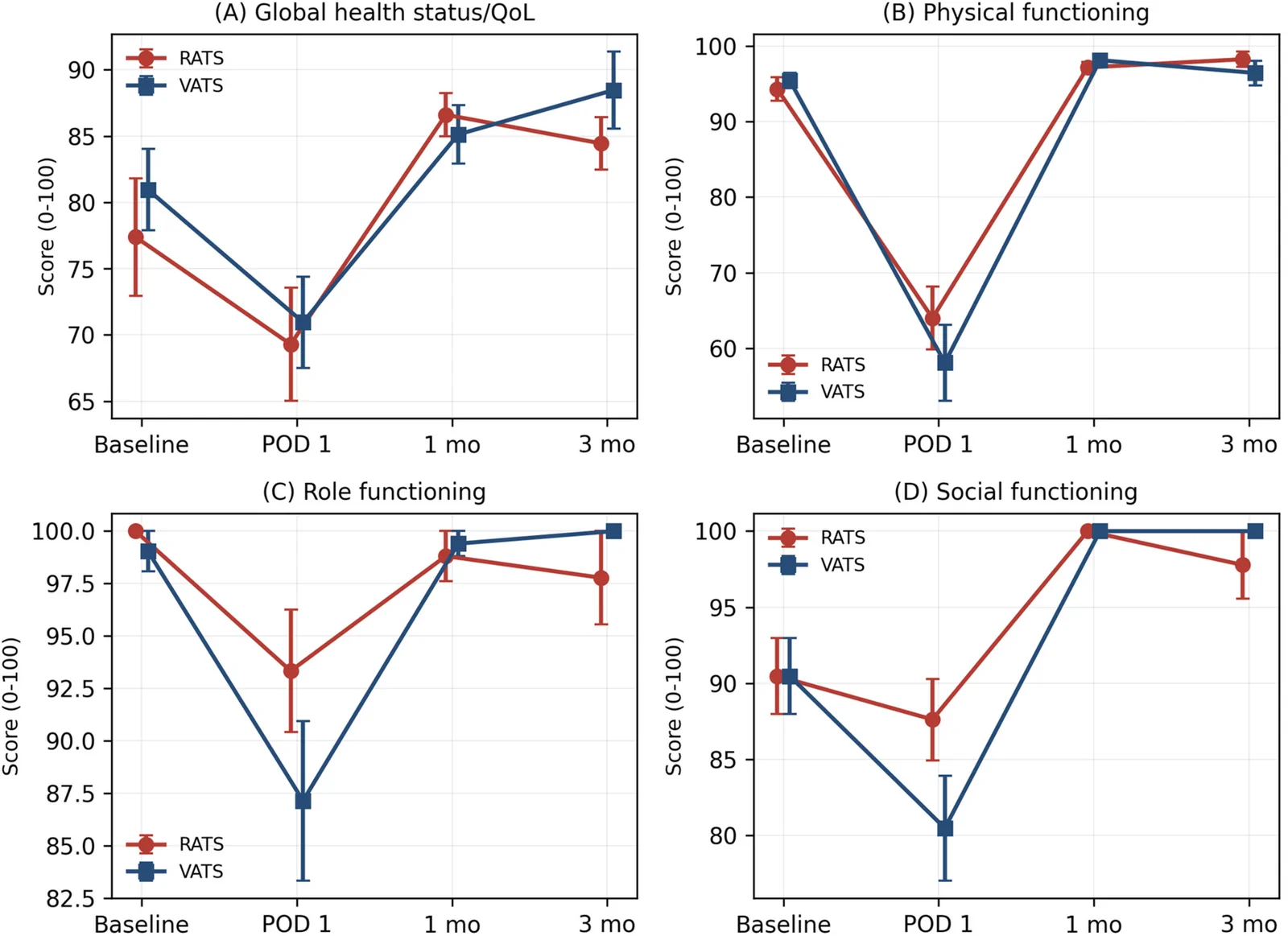
EORTC QLQ-C30 functional and global trajectories in the procedure- and nodal-management-matched cohort. Points are means and error bars are standard errors. Higher scores indicate better functioning.

**Table 3:** Patient-reported outcomes by timepoint.

**Outcome**	**Timepoint**	**n R**	**RATS**	**n V**	**VATS**	**Difference**	**P**
Global health status/QoL	Preoperative	35	77.4 ± 26.2	35	81.0 ± 18.1	−3.6	0.923
Global health status/QoL	POD 1	35	69.3 ± 25.3	35	71.0 ± 20.3	−1.7	0.952
Global health status/QoL	1 month	28	86.6 ± 8.6	28	85.1 ± 11.6	1.5	0.699
Global health status/QoL	3 months	15	84.4 ± 7.6	13	88.5 ± 10.5	−4.0	0.227
Physical functioning	Preoperative	35	94.3 ± 9.2	35	95.4 ± 6.0	−1.1	>0.99
Physical functioning	POD 1	35	64.0 ± 24.5	35	58.1 ± 29.9	5.9	0.412
Physical functioning	1 month	28	97.1 ± 3.8	28	98.1 ± 3.6	−1.0	0.282
Physical functioning	3 months	15	98.2 ± 4.0	13	96.4 ± 5.8	1.8	0.448
Role functioning	Preoperative	35	100.0 ± 0.0	35	99.0 ± 5.6	1.0	0.331
Role functioning	POD 1	35	93.3 ± 17.2	35	87.1 ± 22.5	6.2	0.159
Role functioning	1 month	28	98.8 ± 6.3	28	99.4 ± 3.1	−0.6	>0.99
Role functioning	3 months	15	97.8 ± 8.6	13	100.0 ± 0.0	−2.2	0.390
Emotional functioning	Preoperative	35	83.6 ± 18.5	35	85.7 ± 13.2	−2.1	0.942
Emotional functioning	POD 1	35	86.4 ± 13.9	35	84.0 ± 14.5	2.4	0.376
Emotional functioning	1 month	28	99.7 ± 1.6	28	98.2 ± 5.7	1.5	0.296
Emotional functioning	3 months	15	100.0 ± 0.0	13	100.0 ± 0.0	0.0	>0.99
Social functioning	Preoperative	35	90.5 ± 14.7	35	90.5 ± 14.7	0.0	0.932
Social functioning	POD 1	35	87.6 ± 15.8	35	80.5 ± 20.4	7.1	0.165
Social functioning	1 month	28	100.0 ± 0.0	28	100.0 ± 0.0	0.0	>0.99
Social functioning	3 months	15	97.8 ± 8.6	13	100.0 ± 0.0	−2.2	0.390
Fatigue	Preoperative	35	12.1 ± 12.7	35	12.1 ± 13.3	0.0	>0.99
Fatigue	POD 1	35	25.1 ± 27.5	35	28.3 ± 22.9	−3.2	0.395
Fatigue	1 month	28	1.2 ± 6.3	28	2.0 ± 5.3	−0.8	0.191
Fatigue	3 months	15	1.5 ± 5.7	13	2.6 ± 4.9	−1.1	0.289
Pain (QLQ-C30)	Preoperative	35	5.2 ± 9.7	35	3.8 ± 9.1	1.4	0.421
Pain (QLQ-C30)	POD 1	35	16.7 ± 22.9	35	20.0 ± 21.3	−3.3	0.340
Pain (QLQ-C30)	1 month	28	10.1 ± 9.4	28	8.3 ± 9.6	1.8	0.457
Pain (QLQ-C30)	3 months	15	4.4 ± 7.6	13	2.6 ± 6.3	1.9	0.496
Dyspnoea (QLQ-C30)	Preoperative	35	7.6 ± 14.2	35	9.5 ± 15.3	−1.9	0.592
Dyspnoea (QLQ-C30)	POD 1	35	16.2 ± 21.9	35	20.0 ± 20.1	−3.8	0.324
Dyspnoea (QLQ-C30)	1 month	28	14.3 ± 16.8	28	16.7 ± 17.0	−2.4	0.602
Dyspnoea (QLQ-C30)	3 months	15	6.7 ± 13.8	13	12.8 ± 16.9	−6.2	0.303
Insomnia	Preoperative	35	19.0 ± 24.6	35	15.2 ± 20.4	3.8	0.587
Insomnia	POD 1	35	22.9 ± 23.9	35	21.9 ± 24.2	1.0	0.852
Insomnia	1 month	28	4.8 ± 11.9	28	1.2 ± 6.3	3.6	0.169
Insomnia	3 months	15	0.0 ± 0.0	13	2.6 ± 9.2	−2.6	0.316
NRS pain score	Preoperative	35	0.1 ± 0.3	35	0.3 ± 1.1	−0.2	0.671
NRS pain score	POD 1	35	2.2 ± 1.6	35	2.2 ± 1.9	0.0	0.744
NRS pain score	1 month	28	0.9 ± 0.7	28	0.4 ± 0.6	0.4	0.018
NRS pain score	3 months	15	0.3 ± 0.6	13	0.0 ± 0.0	0.3	0.103
EQ-VAS	Preoperative	35	89.8 ± 9.1	35	90.0 ± 7.5	−0.2	0.801
EQ-VAS	POD 1	35	74.8 ± 14.6	35	70.6 ± 14.4	4.2	0.163
EQ-VAS	1 month	28	88.0 ± 8.2	28	89.8 ± 5.8	−1.8	0.386
EQ-VAS	3 months	15	90.7 ± 6.2	13	89.0 ± 8.3	1.7	0.724
Coughing (LC13)	Preoperative	35	14.3 ± 21.8	35	17.1 ± 18.7	−2.9	0.361
Coughing (LC13)	POD 1	35	19.0 ± 18.6	35	20.0 ± 20.1	−1.0	0.905
Coughing (LC13)	1 month	28	16.7 ± 17.0	28	16.7 ± 19.2	0.0	0.903
Coughing (LC13)	3 months	15	13.3 ± 16.9	13	5.1 ± 12.5	8.2	0.167
Dyspnoea (LC13)	Preoperative	35	10.2 ± 11.9	35	9.2 ± 11.3	1.0	0.767
Dyspnoea (LC13)	POD 1	35	13.7 ± 11.8	35	18.1 ± 16.2	−4.4	0.318
Dyspnoea (LC13)	1 month	28	6.0 ± 10.7	28	5.6 ± 9.8	0.4	0.872
Dyspnoea (LC13)	3 months	15	1.5 ± 3.9	13	0.9 ± 3.1	0.6	0.667
Alopecia (LC13)	Preoperative	35	17.1 ± 24.7	35	10.5 ± 21.0	6.7	0.162
Alopecia (LC13)	POD 1	35	12.4 ± 21.5	35	10.5 ± 17.7	1.9	0.888
Alopecia (LC13)	1 month	28	0.0 ± 0.0	28	0.0 ± 0.0	0.0	>0.99
Alopecia (LC13)	3 months	15	0.0 ± 0.0	13	0.0 ± 0.0	0.0	>0.99
Chest pain (LC13)	Preoperative	35	7.6 ± 14.2	35	10.5 ± 15.7	−2.9	0.428
Chest pain (LC13)	POD 1	35	8.6 ± 14.8	35	18.1 ± 23.4	−9.5	0.066
Chest pain (LC13)	1 month	28	2.4 ± 8.7	28	4.8 ± 11.9	−2.4	0.400
Chest pain (LC13)	3 months	15	0.0 ± 0.0	13	0.0 ± 0.0	0.0	>0.99
Arm/shoulder pain (LC13)	Preoperative	35	15.2 ± 18.7	35	11.4 ± 19.7	3.8	0.281
Arm/shoulder pain (LC13)	POD 1	35	12.4 ± 18.2	35	18.1 ± 24.7	−5.7	0.429
Arm/shoulder pain (LC13)	1 month	27	0.0 ± 0.0	27	0.0 ± 9.2	0.0	>0.99
Arm/shoulder pain (LC13)	3 months	15	0.0 ± 0.0	13	0.0 ± 0.0	0.0	>0.99

Scores are mean ± SD. Difference is RATS minus VATS. Sample sizes vary because of follow-up attrition and item-level missingness. *P* values are unadjusted for multiple comparisons.

### 3.4 Pain, fatigue and disease-specific symptoms

NRS pain was similar on POD 1 (2.2 vs. 2.2, *P* = 0.744) and at 3 months (0.3 vs. 0.0, *P* = 0.103). At 1 month, RATS had a small nominally higher NRS score (0.9 vs. 0.4; mean difference 0.4; *P* = 0.018), whereas the QLQ-C30 pain scale did not differ (10.1 vs. 8.3, *P* = 0.457). Given the small magnitude and multiple comparisons, this isolated finding should not be interpreted as proof of a clinically important difference (Figure 3).

**Figure 3:**
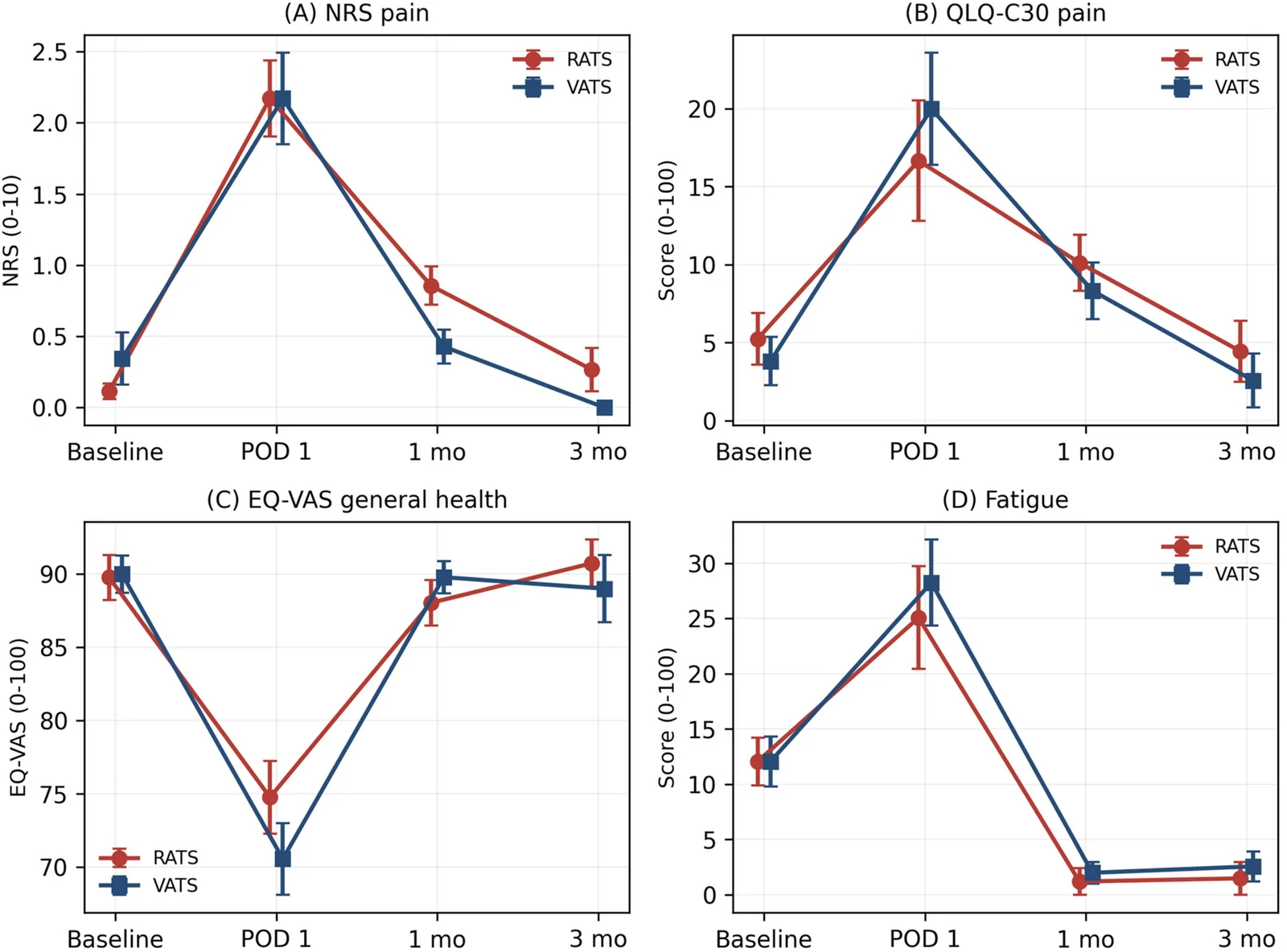
Pain, EQ-VAS and fatigue trajectories. Points are means and error bars are standard errors. Lower NRS, QLQ-C30 pain and fatigue scores indicate less symptom burden; higher EQ-VAS indicates better health.

Fatigue peaked on POD 1 and was low thereafter. At 3 months, fatigue was 1.5 in RATS and 2.6 in VATS (*P* = 0.289), so the apparent increase seen in the previous mixed-procedure analysis was not reproduced. Insomnia was reported because it is a prespecified QLQ-C30 item; no significant between-group difference was observed. QLQ-LC13 cough, dyspnoea and site-specific pain were broadly similar (Figure 4). Alopecia, a standard QLQ-LC13 treatment-related item, was zero in both groups at 1 and 3 months.

**Figure 4:**
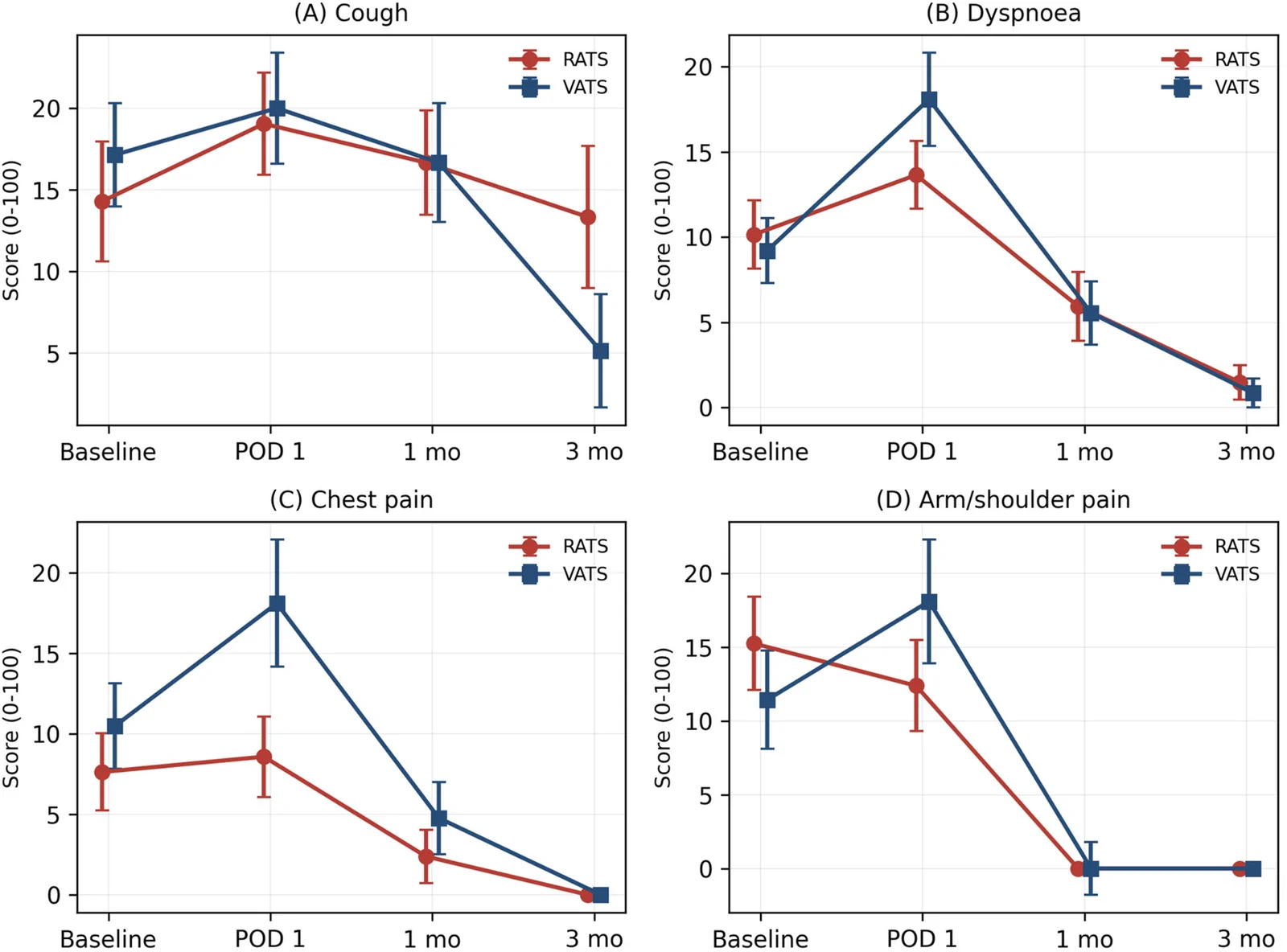
EORTC QLQ-LC13 cough, dyspnoea, chest-pain and arm/shoulder-pain trajectories. Points are means and error bars are standard errors; lower scores indicate less symptom burden.

## 4 Discussion

In this revised, procedure- and nodal-management-matched analysis, short-term patient-reported HRQoL and pain followed broadly similar descriptive trajectories after total-port RATS and uniportal VATS. This comparison addresses the principal limitation of the original analysis, in which wedge, non-parenchymal and more extensive procedures were unevenly distributed. It should nevertheless be interpreted as a small observational comparison, not as evidence of equivalence.

The results are directionally consistent with the RVlob trial, which found no material HRQoL difference between robotic-assisted and video-assisted lobectomy (15), and with early RAVAL health-utility findings (16, 17). Our comparator was uniportal rather than multiportal VATS, and the robotic configuration was completely portal. The present data therefore extend the descriptive evidence to these contemporary configurations while avoiding a claim that incision strategy alone caused the observed outcomes.

The original longer chest-tube duration was attenuated after matching the extent of resection and lymph-node management. In the revised cohort, chest-tube duration did not differ significantly and explicitly recorded air leak was uncommon. The higher drainage volume after RATS, especially for segmentectomy, may reflect unmeasured technical or case-complexity differences, but the available data cannot identify a causal mechanism.

The isolated 1-month NRS difference was small, was not mirrored by the QLQ-C30 pain scale, and did not persist. Likewise, the previously noted 3-month fatigue fluctuation disappeared in the procedure-matched cohort. These examples underscore the instability of nominal secondary findings in a small cohort with later attrition and many comparisons.

### 4.1 Limitations

This study has several limitations. First, it was a single-centre observational study, and matching cannot remove unmeasured confounding. Clinical stage was not separately available, and modest residual imbalance remained in measured covariates. Second, the database recorded treating surgical team but not the individual operating surgeon or surgeon experience; team inclusion in the propensity model does not replace surgeon-level adjustment. Third, the database recorded lymph-node management but not the number or identity of nodal stations, precluding the requested station-count comparison. Fourth, the revised matched cohort contained only 35 patients per group and later questionnaire completion declined to 15 and 13 at 3 months. Fifth, no multiplicity correction was applied, and the study was not designed for equivalence or non-inferiority. The findings are therefore descriptive and hypothesis-generating.

## 5 Conclusions

Among matched patients undergoing the same anatomical resection category and lymph-node management, total-port RATS and uniportal VATS showed broadly similar observed short-term HRQoL and pain trajectories. The small sample, attrition and residual confounding prevent claims of equivalence or causal effects of surgical approach. Larger prospective studies comparing the same operations and incorporating surgeon-level and clinical-stage adjustment are needed.

## Data Availability

The raw data supporting the conclusions of this article will be made available by the authors, without undue reservation.
