# Supplementary material for: Short-term health-related quality of life and pain after total-port robotic versus uniportal video-assisted thoracoscopic anatomical lung resection: a matched prospective cohort study
Source: Front Surg. 2026 Oct 28;13:1919293. doi: 10.3389/fsurg.2026.1919293 (PMC13752135; doi:10.3389/fsurg.2026.1919293)
Supplement: Supplementary file 1 [file Table1.docx]

**Supplementary Table S1**

Complete patient-reported outcome results by timepoint

| **Outcome** | **Timepoint** | **n R** | **RATS** | **n V** | **VATS** | **Difference** | **P** |
| --- | --- | --- | --- | --- | --- | --- | --- |
| Global health status/QoL | Preoperative | 35 | 77.4 ± 26.2 | 35 | 81.0 ± 18.1 | -3.6 | 0.923 |
| Global health status/QoL | POD 1 | 35 | 69.3 ± 25.3 | 35 | 71.0 ± 20.3 | -1.7 | 0.952 |
| Global health status/QoL | 1 month | 28 | 86.6 ± 8.6 | 28 | 85.1 ± 11.6 | 1.5 | 0.699 |
| Global health status/QoL | 3 months | 15 | 84.4 ± 7.6 | 13 | 88.5 ± 10.5 | -4.0 | 0.227 |
| Physical functioning | Preoperative | 35 | 94.3 ± 9.2 | 35 | 95.4 ± 6.0 | -1.1 | >0.99 |
| Physical functioning | POD 1 | 35 | 64.0 ± 24.5 | 35 | 58.1 ± 29.9 | 5.9 | 0.412 |
| Physical functioning | 1 month | 28 | 97.1 ± 3.8 | 28 | 98.1 ± 3.6 | -1.0 | 0.282 |
| Physical functioning | 3 months | 15 | 98.2 ± 4.0 | 13 | 96.4 ± 5.8 | 1.8 | 0.448 |
| Role functioning | Preoperative | 35 | 100.0 ± 0.0 | 35 | 99.0 ± 5.6 | 1.0 | 0.331 |
| Role functioning | POD 1 | 35 | 93.3 ± 17.2 | 35 | 87.1 ± 22.5 | 6.2 | 0.159 |
| Role functioning | 1 month | 28 | 98.8 ± 6.3 | 28 | 99.4 ± 3.1 | -0.6 | >0.99 |
| Role functioning | 3 months | 15 | 97.8 ± 8.6 | 13 | 100.0 ± 0.0 | -2.2 | 0.390 |
| Emotional functioning | Preoperative | 35 | 83.6 ± 18.5 | 35 | 85.7 ± 13.2 | -2.1 | 0.942 |
| Emotional functioning | POD 1 | 35 | 86.4 ± 13.9 | 35 | 84.0 ± 14.5 | 2.4 | 0.376 |
| Emotional functioning | 1 month | 28 | 99.7 ± 1.6 | 28 | 98.2 ± 5.7 | 1.5 | 0.296 |
| Emotional functioning | 3 months | 15 | 100.0 ± 0.0 | 13 | 100.0 ± 0.0 | 0.0 | >0.99 |
| Cognitive functioning | Preoperative | 35 | 91.9 ± 11.0 | 35 | 93.3 ± 10.1 | -1.4 | 0.595 |
| Cognitive functioning | POD 1 | 35 | 94.3 ± 9.9 | 35 | 86.2 ± 17.4 | 8.1 | 0.032 |
| Cognitive functioning | 1 month | 28 | 100.0 ± 0.0 | 28 | 100.0 ± 0.0 | 0.0 | >0.99 |
| Cognitive functioning | 3 months | 15 | 100.0 ± 0.0 | 13 | 100.0 ± 0.0 | 0.0 | >0.99 |
| Social functioning | Preoperative | 35 | 90.5 ± 14.7 | 35 | 90.5 ± 14.7 | 0.0 | 0.932 |
| Social functioning | POD 1 | 35 | 87.6 ± 15.8 | 35 | 80.5 ± 20.4 | 7.1 | 0.165 |
| Social functioning | 1 month | 28 | 100.0 ± 0.0 | 28 | 100.0 ± 0.0 | 0.0 | >0.99 |
| Social functioning | 3 months | 15 | 97.8 ± 8.6 | 13 | 100.0 ± 0.0 | -2.2 | 0.390 |
| Fatigue | Preoperative | 35 | 12.1 ± 12.7 | 35 | 12.1 ± 13.3 | 0.0 | >0.99 |
| Fatigue | POD 1 | 35 | 25.1 ± 27.5 | 35 | 28.3 ± 22.9 | -3.2 | 0.395 |
| Fatigue | 1 month | 28 | 1.2 ± 6.3 | 28 | 2.0 ± 5.3 | -0.8 | 0.191 |
| Fatigue | 3 months | 15 | 1.5 ± 5.7 | 13 | 2.6 ± 4.9 | -1.1 | 0.289 |
| Nausea/vomiting | Preoperative | 35 | 1.9 ± 6.7 | 35 | 1.0 ± 3.9 | 1.0 | 0.636 |
| Nausea/vomiting | POD 1 | 35 | 5.2 ± 9.7 | 35 | 16.2 ± 30.4 | -11.0 | 0.341 |
| Nausea/vomiting | 1 month | 28 | 0.0 ± 0.0 | 28 | 0.6 ± 3.1 | -0.6 | 0.335 |
| Nausea/vomiting | 3 months | 15 | 0.0 ± 0.0 | 13 | 0.0 ± 0.0 | 0.0 | >0.99 |
| Pain (QLQ-C30) | Preoperative | 35 | 5.2 ± 9.7 | 35 | 3.8 ± 9.1 | 1.4 | 0.421 |
| Pain (QLQ-C30) | POD 1 | 35 | 16.7 ± 22.9 | 35 | 20.0 ± 21.3 | -3.3 | 0.340 |
| Pain (QLQ-C30) | 1 month | 28 | 10.1 ± 9.4 | 28 | 8.3 ± 9.6 | 1.8 | 0.457 |
| Pain (QLQ-C30) | 3 months | 15 | 4.4 ± 7.6 | 13 | 2.6 ± 6.3 | 1.9 | 0.496 |
| Dyspnoea (QLQ-C30) | Preoperative | 35 | 7.6 ± 14.2 | 35 | 9.5 ± 15.3 | -1.9 | 0.592 |
| Dyspnoea (QLQ-C30) | POD 1 | 35 | 16.2 ± 21.9 | 35 | 20.0 ± 20.1 | -3.8 | 0.324 |
| Dyspnoea (QLQ-C30) | 1 month | 28 | 14.3 ± 16.8 | 28 | 16.7 ± 17.0 | -2.4 | 0.602 |
| Dyspnoea (QLQ-C30) | 3 months | 15 | 6.7 ± 13.8 | 13 | 12.8 ± 16.9 | -6.2 | 0.303 |
| Insomnia | Preoperative | 35 | 19.0 ± 24.6 | 35 | 15.2 ± 20.4 | 3.8 | 0.587 |
| Insomnia | POD 1 | 35 | 22.9 ± 23.9 | 35 | 21.9 ± 24.2 | 1.0 | 0.852 |
| Insomnia | 1 month | 28 | 4.8 ± 11.9 | 28 | 1.2 ± 6.3 | 3.6 | 0.169 |
| Insomnia | 3 months | 15 | 0.0 ± 0.0 | 13 | 2.6 ± 9.2 | -2.6 | 0.316 |
| Appetite loss | Preoperative | 35 | 7.6 ± 18.2 | 35 | 2.9 ± 9.5 | 4.8 | 0.266 |
| Appetite loss | POD 1 | 35 | 10.5 ± 19.4 | 35 | 18.1 ± 21.9 | -7.6 | 0.096 |
| Appetite loss | 1 month | 28 | 3.6 ± 10.5 | 28 | 0.0 ± 0.0 | 3.6 | 0.081 |
| Appetite loss | 3 months | 15 | 0.0 ± 0.0 | 13 | 2.6 ± 9.2 | -2.6 | 0.316 |
| Constipation | Preoperative | 35 | 10.5 ± 17.7 | 35 | 8.6 ± 14.8 | 1.9 | 0.744 |
| Constipation | POD 1 | 35 | 10.5 ± 17.7 | 35 | 14.3 ± 18.6 | -3.8 | 0.338 |
| Constipation | 1 month | 28 | 1.2 ± 6.3 | 28 | 1.2 ± 6.3 | 0.0 | >0.99 |
| Constipation | 3 months | 15 | 0.0 ± 0.0 | 13 | 0.0 ± 0.0 | 0.0 | >0.99 |
| Diarrhoea | Preoperative | 35 | 2.9 ± 9.5 | 35 | 4.8 ± 11.8 | -1.9 | 0.462 |
| Diarrhoea | POD 1 | 35 | 2.9 ± 9.5 | 35 | 3.8 ± 10.8 | -1.0 | 0.701 |
| Diarrhoea | 1 month | 28 | 2.4 ± 8.7 | 28 | 0.0 ± 0.0 | 2.4 | 0.161 |
| Diarrhoea | 3 months | 15 | 0.0 ± 0.0 | 13 | 0.0 ± 0.0 | 0.0 | >0.99 |
| Financial difficulties | Preoperative | 35 | 10.5 ± 15.7 | 35 | 12.4 ± 24.4 | -1.9 | 0.789 |
| Financial difficulties | POD 1 | 35 | 9.5 ± 17.3 | 35 | 17.1 ± 27.3 | -7.6 | 0.263 |
| Financial difficulties | 1 month | 28 | 0.0 ± 0.0 | 28 | 0.0 ± 0.0 | 0.0 | >0.99 |
| Financial difficulties | 3 months | 15 | 0.0 ± 0.0 | 13 | 0.0 ± 0.0 | 0.0 | >0.99 |
| NRS pain score | Preoperative | 35 | 0.1 ± 0.3 | 35 | 0.3 ± 1.1 | -0.2 | 0.671 |
| NRS pain score | POD 1 | 35 | 2.2 ± 1.6 | 35 | 2.2 ± 1.9 | 0.0 | 0.744 |
| NRS pain score | 1 month | 28 | 0.9 ± 0.7 | 28 | 0.4 ± 0.6 | 0.4 | 0.018 |
| NRS pain score | 3 months | 15 | 0.3 ± 0.6 | 13 | 0.0 ± 0.0 | 0.3 | 0.103 |
| EQ-VAS | Preoperative | 35 | 89.8 ± 9.1 | 35 | 90.0 ± 7.5 | -0.2 | 0.801 |
| EQ-VAS | POD 1 | 35 | 74.8 ± 14.6 | 35 | 70.6 ± 14.4 | 4.2 | 0.163 |
| EQ-VAS | 1 month | 28 | 88.0 ± 8.2 | 28 | 89.8 ± 5.8 | -1.8 | 0.386 |
| EQ-VAS | 3 months | 15 | 90.7 ± 6.2 | 13 | 89.0 ± 8.3 | 1.7 | 0.724 |
| Coughing (LC13) | Preoperative | 35 | 14.3 ± 21.8 | 35 | 17.1 ± 18.7 | -2.9 | 0.361 |
| Coughing (LC13) | POD 1 | 35 | 19.0 ± 18.6 | 35 | 20.0 ± 20.1 | -1.0 | 0.905 |
| Coughing (LC13) | 1 month | 28 | 16.7 ± 17.0 | 28 | 16.7 ± 19.2 | 0.0 | 0.903 |
| Coughing (LC13) | 3 months | 15 | 13.3 ± 16.9 | 13 | 5.1 ± 12.5 | 8.2 | 0.167 |
| Haemoptysis (LC13) | Preoperative | 35 | 1.0 ± 5.6 | 35 | 0.0 ± 0.0 | 1.0 | 0.331 |
| Haemoptysis (LC13) | POD 1 | 35 | 4.8 ± 11.8 | 35 | 3.8 ± 10.8 | 1.0 | 0.731 |
| Haemoptysis (LC13) | 1 month | 28 | 0.0 ± 0.0 | 28 | 0.0 ± 0.0 | 0.0 | >0.99 |
| Haemoptysis (LC13) | 3 months | 15 | 0.0 ± 0.0 | 13 | 0.0 ± 0.0 | 0.0 | >0.99 |
| Dyspnoea (LC13) | Preoperative | 35 | 10.2 ± 11.9 | 35 | 9.2 ± 11.3 | 1.0 | 0.767 |
| Dyspnoea (LC13) | POD 1 | 35 | 13.7 ± 11.8 | 35 | 18.1 ± 16.2 | -4.4 | 0.318 |
| Dyspnoea (LC13) | 1 month | 28 | 6.0 ± 10.7 | 28 | 5.6 ± 9.8 | 0.4 | 0.872 |
| Dyspnoea (LC13) | 3 months | 15 | 1.5 ± 3.9 | 13 | 0.9 ± 3.1 | 0.6 | 0.667 |
| Sore mouth (LC13) | Preoperative | 35 | 1.0 ± 5.6 | 35 | 1.0 ± 5.6 | 0.0 | >0.99 |
| Sore mouth (LC13) | POD 1 | 35 | 2.9 ± 9.5 | 35 | 1.9 ± 11.3 | 1.0 | 0.335 |
| Sore mouth (LC13) | 1 month | 28 | 0.0 ± 0.0 | 28 | 0.0 ± 0.0 | 0.0 | >0.99 |
| Sore mouth (LC13) | 3 months | 15 | 0.0 ± 0.0 | 13 | 0.0 ± 0.0 | 0.0 | >0.99 |
| Dysphagia (LC13) | Preoperative | 35 | 0.0 ± 0.0 | 35 | 1.9 ± 7.9 | -1.9 | 0.160 |
| Dysphagia (LC13) | POD 1 | 35 | 1.9 ± 7.9 | 35 | 1.9 ± 7.9 | 0.0 | >0.99 |
| Dysphagia (LC13) | 1 month | 28 | 0.0 ± 0.0 | 28 | 0.0 ± 0.0 | 0.0 | >0.99 |
| Dysphagia (LC13) | 3 months | 15 | 0.0 ± 0.0 | 13 | 0.0 ± 0.0 | 0.0 | >0.99 |
| Peripheral neuropathy (LC13) | Preoperative | 35 | 2.9 ± 9.5 | 35 | 2.9 ± 9.5 | 0.0 | >0.99 |
| Peripheral neuropathy (LC13) | POD 1 | 35 | 4.8 ± 11.8 | 35 | 5.7 ± 15.1 | -1.0 | 0.969 |
| Peripheral neuropathy (LC13) | 1 month | 28 | 0.0 ± 0.0 | 28 | 0.0 ± 0.0 | 0.0 | >0.99 |
| Peripheral neuropathy (LC13) | 3 months | 15 | 0.0 ± 0.0 | 13 | 0.0 ± 0.0 | 0.0 | >0.99 |
| Alopecia (LC13) | Preoperative | 35 | 17.1 ± 24.7 | 35 | 10.5 ± 21.0 | 6.7 | 0.162 |
| Alopecia (LC13) | POD 1 | 35 | 12.4 ± 21.5 | 35 | 10.5 ± 17.7 | 1.9 | 0.888 |
| Alopecia (LC13) | 1 month | 28 | 0.0 ± 0.0 | 28 | 0.0 ± 0.0 | 0.0 | >0.99 |
| Alopecia (LC13) | 3 months | 15 | 0.0 ± 0.0 | 13 | 0.0 ± 0.0 | 0.0 | >0.99 |
| Chest pain (LC13) | Preoperative | 35 | 7.6 ± 14.2 | 35 | 10.5 ± 15.7 | -2.9 | 0.428 |
| Chest pain (LC13) | POD 1 | 35 | 8.6 ± 14.8 | 35 | 18.1 ± 23.4 | -9.5 | 0.066 |
| Chest pain (LC13) | 1 month | 28 | 2.4 ± 8.7 | 28 | 4.8 ± 11.9 | -2.4 | 0.400 |
| Chest pain (LC13) | 3 months | 15 | 0.0 ± 0.0 | 13 | 0.0 ± 0.0 | 0.0 | >0.99 |
| Arm/shoulder pain (LC13) | Preoperative | 35 | 15.2 ± 18.7 | 35 | 11.4 ± 19.7 | 3.8 | 0.281 |
| Arm/shoulder pain (LC13) | POD 1 | 35 | 12.4 ± 18.2 | 35 | 18.1 ± 24.7 | -5.7 | 0.429 |
| Arm/shoulder pain (LC13) | 1 month | 27 | 0.0 ± 0.0 | 27 | 0.0 ± 9.2 | 0.0 | >0.99 |
| Arm/shoulder pain (LC13) | 3 months | 15 | 0.0 ± 0.0 | 13 | 0.0 ± 0.0 | 0.0 | >0.99 |
| Other pain (LC13) | Preoperative | 25 | 1.3 ± 6.7 | 30 | 0.0 ± 0.0 | 1.3 | 0.290 |
| Other pain (LC13) | POD 1 | 28 | 0.0 ± 0.0 | 27 | 0.0 ± 0.0 | 0.0 | >0.99 |
| Other pain (LC13) | 1 month | 28 | 0.0 ± 0.0 | 25 | 0.0 ± 0.0 | 0.0 | >0.99 |
| Other pain (LC13) | 3 months | 15 | 0.0 ± 0.0 | 13 | 0.0 ± 0.0 | 0.0 | >0.99 |

*Scores are mean ± SD. Difference is RATS minus VATS. P values are unadjusted for multiple comparisons.*
